# Risk and prognostic factors for endometrial carcinoma after diagnosis of breast or Lynch‐associated cancers—A population‐based analysis

**DOI:** 10.1002/cam4.1890

**Published:** 2018-11-28

**Authors:** Sharon E. Johnatty, Colin J. R. Stewart, Deborah Smith, Daniel Buchanan, Yee Leung, Martin K. Oehler, Alison Brand, Penelope M. Webb, Amanda B. Spurdle

**Affiliations:** ^1^ Department of Genetics and Computational Biology QIMR Berghofer Medical Research Institute Brisbane Queensland Australia; ^2^ Department of Histopathology King Edward Memorial Hospital Perth Western Australia Australia; ^3^ School of Woman's and Infants' Health University of Western Australia Perth Western Australia Australia; ^4^ Department of Pathology The Mater Hospital Brisbane Queensland Australia; ^5^ Colorectal Oncogenomics Group, Genetic Epidemiology Laboratory, Department of Pathology The University of Melbourne Parkville Victoria Australia; ^6^ Centre for Epidemiology and Biostatistics, Melbourne School of Population and Global Health The University of Melbourne Melbourne Victoria Australia; ^7^ Genetic Medicine and Family Cancer Clinic Royal Melbourne Hospital Parkville Victoria Australia; ^8^ Department of Gynaecological Oncology King Edward Memorial Hospital Perth Western Australia Australia; ^9^ Department of Gynaecological Oncology Royal Adelaide Hospital Adelaide South Australia Australia; ^10^ Department of Gynaecological Oncology Westmead Hospital, University of Sydney Westmead New South Wales Australia; ^11^ Department of Population Health QIMR Berghofer Medical Research Institute Brisbane Queensland Australia

**Keywords:** endometrial cancer, MMR status, prior cancer, prognosis, risk

## Abstract

We hypothesized that endometrial carcinoma (EC) patients with a prior cancer diagnosis, after accounting for EC arising after tamoxifen‐treated prior breast carcinoma, are more likely to have an underlying genetic basis. We used information from a population‐based study to compare measured risk factors, tumor characteristics, survival, and known mismatch repair (MMR) pathogenic variant status for EC subgroups according to prior diagnosis of cancer (none, breast cancer tamoxifen‐treated or not, Lynch Syndrome (LS)‐associated cancer). Family history of any cancer was increased for EC cases with prior breast cancer, both tamoxifen treated (*P* = 0.005) and untreated (*P* = 0.01). EC cases with prior LS‐associated cancer more often reported family history of LS‐associated cancer (*P* = 0.04) and breast cancer (*P* = 0.05). EC patients with a germline pathogenic MMR gene variant were more likely to report a prior cancer than cases with a MMR proficient tumor (*P* = 0.0001), but more than half (54.5%) of MMR carriers reported no prior cancer. Women developing EC after tamoxifen treatment for breast cancer were significantly more likely to develop EC of malignant mixed mullerian tumor subtype (13.2% vs 2.6%, *P* = 1.3 × 10^−6^), present with stage IV disease (8.8% vs 1.2%, *P* = 1.6 × 10^−6^), and have poorer survival (HR_adj_ 1.96; *P* = 0.001). While report of prior cancer is an indicator of MMR pathogenic variant status, molecular analysis of all ECs at diagnosis is warranted to detect all patients with LS. Results also indicate the importance of longer‐term monitoring of women treated with tamoxifen for symptoms of EC, and the need for studies assessing the biological mechanism underlying the poorer prognosis of this subset of EC patients.

## 1 INTRODUCTION

Endometrial carcinoma (EC) is the fifth most common cancer in women in developed countries, accounting for 4.8% of new cancers and 2.1% of cancer deaths. The highest incidence rates in 2012 were estimated to be 19.1 and 15.6 per 100 000 in North America and Western Europe, respectively,^1^, ^2^ attributed to the greater overall prevalence of obesity and metabolic syndromes in these regions.^3^

Established nongenetic risk factors for EC include age and exposure to exogenous estrogens, or endogenous hyperestrogenic status associated with nulliparity, early age at menarche, late‐onset menopause and obesity.^4^ In addition, tamoxifen use for treatment of invasive breast cancer is associated with an increased risk of developing EC,^5^ with several reports indicating that EC arising after tamoxifen‐treated breast cancer may have poorer prognostic features.^6^, ^7^ Genetic risk factors for EC have been supported by the fact that there is at least a twofold increased risk of EC among women with at least one first‐ or second‐degree relative with EC.^12^, ^13^ Such genetic factors include high‐risk pathogenic variants in the DNA mismatch repair (MMR) genes causing Lynch Syndrome, and very rarely, germline loss‐of‐function variants in the *PTEN* tumor suppressor gene causing Cowden Syndrome.^14^ We have recently shown that carriage of a pathogenic variant in an MMR gene only partly accounts for risk of EC associated with reported family history,^12^ indicating that additional genetic risk factors remain to be identified. It is clear that common genetic variants identified through large‐scale genomewide association studies and candidate gene studies also contribute to EC risk, with currently identified risk variants accounting for ~6.8% of the familial relative risk of EC, and modeling studies suggesting an upper estimate of 28% of familial relative risk may be due to common variants.^15^, ^16^

There is evidence that individuals with moderate‐high‐risk pathogenic variants in cancer predisposition genes are more likely to develop multiple cancers in their lifetime. The cumulative risk of metachronous colorectal carcinoma was reported to be up to 69% among carriers of pathogenic germline MMR gene variants.^23^ Members of Li Fraumeni families (due to *TP53* pathogenic variants) were shown to have a fivefold increased risk of developing a second primary cancer compared to the general population.^24^ Further, studies evaluating *BRCA1/2* pathogenic variant carriers have reported that women with a first diagnosis of breast carcinoma had a significantly higher risk of developing a pathologically similar contralateral breast carcinoma^25^ or ovarian carcinoma compared to noncarriers.^26^

We thus hypothesized that report of a prior cancer, after accounting for EC arising after tamoxifen‐treated prior breast carcinoma, might identify women with EC more likely to have an underlying genetic basis. We anticipated that EC patients with a prior cancer diagnosis might be more likely to report a family history of cancer than EC cases with no prior cancer diagnosis. In addition, we considered the possibility that classical epidemiological risk factors for EC (such as obesity) may be less important for EC patients with prior cancer report (and suspected genetic basis). To investigate these questions, we detailed prior cancer report and tamoxifen use for women with EC and controls participating in the population‐based Australian National Endometrial Cancer Study (ANECS). We compared epidemiological risk factors, reported family history, tumor characteristics, survival, and known MMR gene pathogenic variant status for EC subgroups according to cancer types diagnosed prior to recruitment, and for women without EC and no reported personal history of cancer.

## 2 METHODS

### 2.1 Study sample sets

All ANECS participants provided informed written consent, and approval was obtained from the QIMR Berghofer Medical Research Institute Human Research Ethics Committee, participating hospitals and cancer registries. Details of participant ascertainment, eligibility criteria, questionnaires, and data collection, including blood samples for genetic testing, and assessment of family history have been previously reported.^12^, ^27^ Clinicopathological data including histological subtype, grade, tumor stage, and lymphovascular space invasion (LVSI) were abstracted from medical/pathology records. Cases were re‐staged using the International Federation of Gynecology and Obstetrics (FIGO) 2009 criteria. Following convention regarding grading of “non‐endometrioid” tumors, and knowledge of prognostic features of tumors of mixed histology,^28^ tumor histology and grade were combined in a single variable with the following categories: endometrioid grade 1, endometrioid grade 2, endometrioid grade 3, serous (≥5%), clear cell (≥10%, and no serous ≥5%), carcinosarcoma (malignant mixed mullerian tumor, MMMT), and other epithelial. Vital status was determined from medical records and using probabilistic record data linkage to the Australian National Death Index. Survival time was calculated from date of primary treatment for EC to date of death (overall, EC‐specific) or censored at 31 December 2013.

The dataset used for this analysis was based on information for 1399 EC cases and 740 controls who were eligible to participate. Information about report of prior cancer was extracted from questionnaires (cases and controls), and supplemented by information from medical reports ascertained by clinical follow‐up studies, and from information about prior cancer noted in endometrial cancer pathology reports (cases only). Tamoxifen use for individuals with prior breast cancer was verified where possible from breast or EC pathology reports, and/or from clinical records.

The detailed breakdown of all prior cancers in controls and EC cases is reported in Table S1. After in‐depth reviews from all available data sources, we identified 86 controls and 184 EC cases with a prior cancer diagnosis. The most commonly reported prior cancer diagnosis was breast cancer; 41 controls and 101 EC cases (97 self‐reported and four identified from medical records). Among the 101 EC cases with prior breast cancer, 68 had been treated with tamoxifen (47 self‐reported, and another 21 identified from clinical follow‐up studies or pathology reports), and 33 self‐reported no tamoxifen use with no contradictory information from all available clinical records.

We also analyzed a subset of EC cases with at least one diagnosis of a cancer type falling into the Lynch Syndrome (LS) spectrum (termed LS‐associated) prior to EC diagnosis. Cancers considered to be LS‐associated included cancer of the bile duct, bladder, brain, colon/rectum, duodenum, endometrium, gastrointestinal tract, ovary, pancreas, renal pelvis, and stomach. Of 15 women self‐reporting prior ovarian cancer, only two were determined to be true prior cancers, based on age at diagnosis and information from clinical follow‐up or pathology reports; for 11 women, their ovarian cancer diagnosis was actually concurrent with EC, and EC pathology reports for another two women indicated that their ovaries were normal and intact at hysterectomy for EC. For the purposes of this analysis, women who had ovarian cancer that was concurrent with EC were included among those with no prior cancer unless they had been diagnosed with another cancer prior to their EC diagnosis. A total of 27 EC cases had been diagnosed with at least one prior LS‐associated cancer.

In addition to breast and LS‐associated cancers, there were 68 EC patients and 38 controls with reports of other cancer types, with some individuals reporting multiple prior cancer types (Table S1). The remaining 1215 EC patients and 653 controls reported no prior cancer at enrollment in ANECS. An additional control had insufficient data to determine her prior cancer status.

### 2.2 Statistical analysis

The risk of EC associated with known epidemiologic risk factors was evaluated using age‐adjusted logistic regression models. Using controls reporting no prior cancer at interview (n = 653) as reference, risk estimates were evaluated for: (a) cases with no history of cancer prior to EC diagnosis (n = 1215); (b) cases diagnosed with breast cancer prior to EC who were treated with tamoxifen (n = 68); (c) cases diagnosed with breast cancer prior to EC with no known tamoxifen use (n = 33); and (d) cases who had been diagnosed with at least one LS‐associated cancer prior to EC (n = 27). Age (at diagnosis for women with EC, at enrolment for controls) was entered into regression models as a continuous variable. Body mass index (BMI) was analyzed as a categorical variable comparing women with a BMI 25‐29, and ≥30 to those with BMI <25 kg/m^2^. Parity, defined as the number of pregnancies ≥6 months, was analyzed as 1 or ≥2 vs 0. Age at menarche was analyzed as 12‐13 or ≥14 vs ≤11. Risk factors for EC, for example, oral contraceptive use, smoking, and ≥3 months use of systemic menopausal hormone therapy (postmenopausal women only) were entered into models as ever vs never use. We also evaluated all patient subgroups for EC risk associated with family cancer history comparing any vs no first‐ or second‐degree relative (FDR or SDR) reporting any invasive cancer, any LS cancer, and breast or ovarian cancer. For patient characteristics analyzed as ordinal variables, we assessed the trend across ordered groups using the nonparametric Cuzick's test for trend.

We used Cox regression models to evaluate overall and EC‐specific survival of cases with a prior breast cancer diagnosis and tamoxifen use, prior breast cancer and no known tamoxifen use, and cases diagnosed with at least one LS‐associated cancer prior to EC, compared to those with no history of cancer prior to EC diagnosis. Cox models were adjusted for the following: age at EC diagnosis as a continuous variable; tumor stage in three categories (FIGO stages I, II, and III & IV); tumor histology in seven categories (endometrioid Grade 1, 2, or 3, serous, clear cell, carcinosarcoma/MMMT, other epithelial); presence/absence of LVSI. Survival time was defined as the interval from date of first treatment for EC to date of death from any cause or due to EC, or censored at 31 December 2013.

In addition, we evaluated the relationship between prior cancer and tumor/germline MMR status as previously determined^27^ for 722 of the 1399 cases included in this study; 558 were classified as IHC proficient and did not undergo germline genetic testing on the assumption they were very unlikely to carry a pathogenic MMR gene variant, 142 were IHC deficient with no pathogenic variant identified by germline DNA testing, and 22 were IHC deficient and carriers of a pathogenic MMR gene variant. Differences in the frequency of IHC proficient and deficient tumors were evaluated for patients with prior breast cancer according to tamoxifen treatment, and patients with prior LS‐associated cancer compared to patients with no prior cancer.

All tests for association were two‐tailed, and performed using STATA SE v. 13 (Stata Corp., USA), and the R project for Statistical Computing version 3.2.2 (http://www.r-project.org/).

## 3 RESULTS

As detailed in Table S1, there was no indication that report of prior cancer was elevated in patients compared to controls, overall (13.2% vs 11.8%, *P* = 0.4), or considering individual cancer types. The proportion with reported tamoxifen‐treated breast cancer was nonsignificantly higher in cases compared to controls (3.4% vs 2.8% based on self‐reported data only, *P* = 0.5; additional exploration of tamoxifen usage for cases only (from pathology reports or clinical follow‐up) increased the proportion of tamoxifen‐treated prior breast cancer among cases to 4.9%). There was no difference between cases and controls for report of prior LS‐associated cancers overall (1.9% vs 2.0%). There was some suggestion that cases were more likely to report two or more prior cancer types compared to controls (1.5% vs 0.7%), but this difference was not statistically significant (*P* = 0.1).

### 3.1 Association with epidemiological risk factors

Results are detailed in Table 1. Comparison of age at endometrial cancer diagnosis in patient subgroups shows that patients with prior breast cancer were diagnosed with EC at somewhat older age than those with no prior cancer (mean age 67 and 66 years among tamoxifen users and nonusers, respectively, vs 61 years among those with no prior cancers; *P* ≤ 0.002). There was no evidence that endometrial cancer was diagnosed at an earlier age for patients with prior LS‐associated cancers (62 years, *P* = 0.4).

**Table 1:** Association of epidemiological risk factors with endometrial cancer risk, according to report of prior breast or lynch syndrome cancer

Characteristics^a^	Controls with no prior cancer (n = 653)	Patients with no prior Cancer (n = 1215)			Patients with prior breast cancer & tamoxifen use (n = 68)			Patients with prior breast cancer and no tamoxifen use (n = 33)			Patients with prior LS‐associated cancers (n = 27)		
	N^b^ (%)	N^b^ (%)	OR^c^ (95% CI)	*P* d	N^b^ (%)	OR^c^ (95% CI)	*P* d	N^b^ (%)	OR^c^ (95% CI)	*P* d	N^b^ (%)	OR^c^ (95% CI)	*P* d
Age (years)													
Mean age (SD)	60.6 (9.9)	60.9 (9.4)		0.5	66.8 (8.3)		<0.0001	66.0 (7.4)		0.002	62.1 (10.0)		0.4
Age Range	31.5‐80.1	26.4‐80.0			47.0‐78.7			51.2‐78.4			33.3‐78.4		
<50	94 (14.4)	142 (11.7)			3 (4.4)			0 (0.0)			2 (7.4)		
≥ 50	559 (85.6)	1073 (88.3)			65 (95.6)			33 (100.0)			25 (92.6)		
BMI													
<25	315 (49.5)	295 (24.4)	1.00		23 (33.8)	1.00		12 (36.4)	1.00		7 (25.9)	1.00	
25‐29.9	189 (29.7)	296 (24.5)	1.65 (1.29‐2.10)	5.58E−05	25 (36.8)	1.63 (0.89‐3.00)	0.05	9 (27.3)	1.14 (0.47‐2.78)	0.77	10 (37.0)	2.32 (0.87‐6.21)	0.09
≥30	133 (20.9)	617 (51.1)	4.96 (3.88‐6.35)	2.12E−37	20 (29.4)	2.24 (1.17‐4.29)	0.02	12 (36.4)	2.47 (1.07‐5.70)	0.03	10 (37.0)	3.38 (1.26‐9.07)	0.02
Test for Trend				1.14E−39			0.01			0.06			0.01
OC use													
Never	106 (16.3)	381 (31.4)	1.00		27 (39.7)	1.00		14 (42.4)	1.00		10 (37.0)	1.00	
Ever	546 (83.7)	833 (68.6)	0.41 (0.32‐0.53)	1.92E−12	41 (60.3)	0.45 (0.25‐0.81)	0.008	19 (57.6)	0.36 (0.16‐0.78)	0.01	17 (63.0)	0.33 (0.14‐0.78)	0.01
Parity													
0	46 (7.0)	217 (17.9)	1.00		9 (13.2)	1.00		6 (18.2)	1.00		3 (11.1)	1.00	
1	59 (9.0)	119 (9.8)	0.42 (0.27‐0.66)	1.39E−04	8 (11.8)	0.69 (0.24‐2.01)	0.5	4 (12.1)	0.51 (0.13‐1.97)	0.33	4 (14.8)	1.02 (0.22‐4.78)	0.98
≥2	548	879 (72.3)	0.32 (0.23‐0.45)	7.52E−11	51 (75.0)	0.40 (0.18‐0.90)	0.03	23 (69.7)	0.26 (0.10‐0.69)	0.01	20 (74.1)	0.51 (0.15‐1.82)	0.30
Test for Trend				1.36E−10			0.05			0.03			0.18
Smoking													
Never	384 (58.9)	785 (64.6)	1.00		44 (64.7)	1.00		25 (78.1)	1.00		20 (74.1)	1.00	
Ever	268 (41.1)	430 (35.4)	0.79 (0.65‐0.96)	0.02	24 (35.3)	0.95 (0.56‐1.63)	0.86	7 (21.9)	0.46 (0.20‐1.09)	0.08	7 (25.9)	0.52 (0.21‐1.25)	0.14
Age at menarche													
≤11	96 (15.9)	269 (22.3)	1.00		18 (26.5)	1.00		4 (12.5)	1.00		7 (25.9)	1.00	
12‐13	328 (50.9)	584 (48.5)	0.64 (0.49‐0.83)	0.001	30 (44.1)	0.50 (0.26‐0.96)	0.04	19 (59.4)	1.44 (0.47‐4.37)	0.52	10 (37.0)	0.42 (0.15‐1.13)	0.09
14+	221 (34.3)	352 (29.2)	0.57 (0.42‐0.75)	1.04E−04	20 (29.4)	0.47 (0.23‐0.93)	0.03	9 (28.1)	0.99 (0.29‐3.31)	0.98	9 (33.3)	0.56 (0.20‐1.54)	0.26
Test for Trend				0.0002			0.08			0.70			0.47
Hormone use in postmenopausal women													
Never	298 (57.6)	643 (67.4)	1.00		45 (69.2)	1.00		20 (60.6)	1.00		14 (60.9)	1.00	
Ever	219 (42.4)	311 (32.6)	0.66 (0.53‐0.82)	1.91E−04	20 (30.8)	0.62 (0.36‐1.09)	0.10	13 (39.4)	0.89 (0.43‐1.83)	0.75	9 (39.1)	0.88 (0.37‐2.06)	0.76

a Age at diagnosis for patients, at interview for controls; SD, standard deviation; BMI, body mass index; OC, oral contraceptive use.

b Ns may not sum to the total because of missing or unknown data; proportions (%) sum to 100% of observations where data available and excludes missing/unknowns.

c Risk estimates (Odds Ratios and 95% confidence intervals) are adjusted for age as a continuous variable (at diagnosis for patients, at interview for controls).

d *P*‐values for Mean age variables represent pairwise comparisons of means between Controls no prior cancer and Case subgroups.

Endometrial carcinoma risk in women with no cancer prior to EC diagnosis was associated with known epidemiologic risk factors for this disease.^4^ Compared to controls with no prior history of cancer, BMI ≥ 30 was associated with an almost fivefold increased risk of EC for women with no prior cancer history. There was a highly significant trend in increased risk associated with BMI categories of ≥25 to <30 and ≥30 (*P* = 1.4 × 10^−39^). Oral contraceptive use, older age at menarche, parity, and use of systemic hormone therapy (mostly combination therapy in postmenopausal women) were inversely associated with EC risk in women with no prior cancers (*P* ≤ 0.001), and there was a significant trend toward a lower risk of EC associated with older age at menarche (*P* = 0.0002) and higher numbers of full‐term births (*P* = 1.4 × 10^−10^; Table 1). In addition, there was evidence to support the previously reported inverse association of EC risk with smoking (*P* = 0.02). The risk estimates for EC following a prior breast or LS‐associated cancer associated with the risk factors highlighted above were necessarily imprecise given the smaller sample sizes. Nevertheless, they were generally in the same direction as those reported for EC with no prior cancer, with elevated risk associated with increasing BMI, and decreased risk associated with OC use, increasing parity, later age at menarche, and postmenopausal HRT use. Possible exceptions were ever‐smoking, which did not appear to be inversely associated with EC among women with prior breast cancer and tamoxifen use, while age at menarche did not appear to be inversely associated with EC among women with prior breast cancer and no tamoxifen use.

### 3.2 Association with reported family history of cancer

As shown in Table 2, women with no prior cancer had a 43% increased risk of EC if at least one FDR or SDR was reported to have any cancer diagnosis (*P* = 2.5 × 10^−4^) or any LS cancer (*P* = 2.7 × 10^−4^), but no significant increased EC risk associated with having an FDR or SDR with breast cancer (*P* = 0.3) or ovarian cancer (*P* = 0.9). Despite small sample sizes, there was marginal evidence that women with prior breast cancer had increased risk of EC if they reported a family history of cancer in close relatives, be that family history of any cancer type (OR 2.23, *P* = 0.005 for tamoxifen users; OR 2.80, *P* = 0.01 for nonusers), family history of breast cancer (OR 1.98, *P* = 0.01 for tamoxifen users; OR 1.95, *P* = 0.07 for nonusers), or family history of LS‐associated cancers (OR 1.78, *P* = 0.03 for tamoxifen users). All cases with prior LS‐associated cancer reported a family history of cancer, and for this subgroup, there was evidence for increased risk associated with reported family history of LS‐associated cancer (OR 2.34, *P* = 0.04) or family history of breast cancer (OR 2.15, *P* = 0.05).

**Table 2:** Association of reported family history of cancer with endometrial cancer risk, according to report of prior breast or lynch syndrome cancer

Family Cancer History	Controls with no prior cancer (n = 653)	Patients with no prior Cancer (n = 1215)			Patients with prior breast cancer and tamoxifen use (n = 68)			Patients with prior breast cancer and no tamoxifen use (n = 33)			Patients with prior LS‐associated cancers (n = 27)		
	N^a^ (%)	N^a^ (%)	OR^b^ (95% CI)	*P*	N^a^ (%)	OR^b^ (95% CI)	*P*	N^a^ (%)	OR^b^ (95% CI)	*P*	N^a^ (%)	OR^b^ (95% CI)	*P*
Family history any cancer (FDR &/or SDR)^c^													
No	297 (45.5)	447 (36.8)	1.00		19 (27.9)	1.00		8 (24.2)	1.00		0 (0.0)	1.00	
Yes	356 (54.5)	768 (63.2)	1.43 (1.18‐1.74)	2.52E‐04	49 (72.1)	2.23 (1.27‐3.90)	0.005	25 (75.8)	2.80 (1.23‐6.34)	0.014	27 (100.0)	na	na
FDR &/or SDR with any Lynch‐ associated cancer^d^													
No	376 (57.6)	593 (48.8)	1.00		30 (44.1)	1.00		18 (54.5)	1.00		10 (37.0)	1.00	
Yes	277 (42.4)	622 (51.2)	1.43 (1.18‐1.73)	2.73E‐04	38 (55.9)	1.78 (1.06‐2.97)	0.03	15 (45.5)	1.21 (0.59‐2.46)	0.598	17 (63.0)	2.34 (1.05‐5.19)	0.037
FDR &/or SDR with breast cancer													
No	477 (73.0)	859 (70.8)	1.00		38 (56.7)	1.00		19 (57.6)	1.00		15 (55.6)	1.00	
Yes	176 (27.0)	355 (29.2)	1.12 (0.90‐1.38)	0.31	29 (43.3)	1.98 (1.17‐3.34)	0.011	14 (42.4)	1.95 (0.95‐4.01)	0.068	12 (44.4)	2.15 (0.99‐4.69)	0.054
Missing		1			1								
FDR &/SDR with ovarian cancer													
No	627 (96.0)	1164 (95.9)	1.00		66 (98.5)	1.00		32 (97.0)	1.00		27 (100.0)	1.00	
Yes	26 (4.0)	50 (4.1)	1.03 (0.64‐1.68)	0.89	1 (1.5)	0.36 (0.05‐2.73)	0.32	1 (3.0)	0.74 (0.10‐5.64)	0.768	0 (0.0)	na	na
Missing	0	1			1								

a Ns may not sum to the total because of missing or unknown data; proportions (%) sum to 100% of observations where data available and excludes missing/unknowns.

b ORs are adjusted for age as a continuous variable (at diagnosis for patients and at interview for controls).

c Family history of cancer reported in at least one first‐ or second‐degree relative.

d Report of at least one first‐ or second‐degree relative with bile duct, bladder, brain, colon/rectum, duodenal, endometrial, gastrointestinal/GI, ovary/FT, pancreas, renal pelvis, stomach cancers.

### 3.3 Differences in EC tumor pathology prognostic variables and survival according to prior cancer

Cases with prior breast cancer (irrespective of tamoxifen treatment) or prior LS‐associated cancer were less likely to present with a grade 1 or 2 endometrioid adenocarcinoma than the reference group of EC cases with no prior cancer (Table 3). In particular, women with tamoxifen‐treated breast cancer were significantly more likely to develop an endometrial carcinosarcoma (MMMT; 13.2% vs 2.6%, *P* = 1.3 × 10^−6^), and present with stage IV disease (8.8% vs 1.2%, *P* = 1.6 × 10^−6^). In accordance with this observation, women with tamoxifen‐treated breast cancer had poorer overall survival (HR_adj_ 1.96; *P* = 0.001) and EC‐specific survival (HR_adj_ 1.91; *P* = 0.02) compared to women with no prior cancer. Women with prior breast cancer and no tamoxifen use or prior LS‐associated cancer exhibited similar survival to those with no prior cancer (*P* ≥ 0.27; Table 4 & Figure S1). The minimum follow‐up time was 7.8 years post‐treatment.

**Table 3:** Endometrial tumor pathology prognostic characteristics according to report of prior breast or Lynch Syndrome‐associated cancer

Tumor characteristic	Patients with no prior Cancer (n = 1215)	Patients with prior breast cancer and tamoxifen use (n = 68)			Patients with prior breast cancer and no tamoxifen use (n = 33)			Patients with prior LS‐associated cancers (n = 27)		
	N (%)	N (%)	OR^a^ (95% CI)	*P*_chi‐sq_	N (%)	OR^a^ (95% CI)	*P*_chi‐sq_	N (%)	OR^a^ (95% CI)	*P*_chi‐sq_
Tumor histology and grade										
Endometrioid grade 1	637 (52.4)	31 (45.6)	0.76 (0.45‐1.28)	0.27	17 (51.5)	0.96 (0.45‐2.06)	0.92	11 (40.7)	0.62 (0.26‐1.45)	0.23
Endometrioid grade 2	315 (25.9)	8 (11.8)	0.38 (0.16‐0.81)	0.0088	7 (21.2)	0.77 (0.28‐1.84)	0.54	5 (18.5)	0.65 (0.19‐1.78)	0.38
Endometrioid grade 3	100 (8.2)	7 (10.3)	1.28 (0.48‐2.90)	0.55	3 (9.1)	1.12 (0.21‐3.68)	0.86	3 (11.1)	1.39 (0.26‐4.71)	0.59
Serous (>5%)	89 (7.3)	9 (13.2)	1.93 (0.81‐4.08)	0.07	4 (12.1)	1.75 (0.44‐5.13)	0.30	5 (18.5)	2.88 (0.83‐8.02)	0.03
Clear cell (>10%), no serous	29 (2.4)	2 (2.9)	1.23 (0.14‐5.08)	0.80	2 (6.1)	2.64 (0.29‐11.24)	0.18	1 (3.7)	1.57 (0.04‐10.28)	0.66
Carcinosarcoma (MMMT)	32 (2.6)	9 (13.2)	5.64 (2.26‐12.77)	1.30E‐06	0 (0.0)	na	na	2 (7.4)	2.96 (0.33‐12.73)	0.13
Other epithelial^b^	14 (1.2)	2 (2.9)	2.60 (0.28‐11.68)	0.20						
FIGO stage										
I	1011 (83.2)	53 (77.9)	0.72 (0.39‐1.41)	0.28	24 (72.7)	0.54 (0.24‐1.34)	0.11	22 (81.5)	0.89 (0.32‐3.04)	0.81
II	88 (7.2)	2 (2.9)	0.39 (0.05‐1.50)	0.18	4 (12.1)	1.77 (0.44‐5.19)	0.29	2 (7.4)	1.02 (0.12‐4.22)	0.97
III	95 (7.8)	7 (10.3)	1.35 (0.51‐3.07)	0.46	3 (9.1)	1.18 (0.23‐3.90)	0.79	2 (7.4)	0.94 (0.11‐3.88)	0.94
IV	15 (1.2)	6 (8.8)	7.74 (2.37‐21.95)	1.59E‐06	1 (3.0)	2.50 (0.06‐17.23)	0.37	1 (3.7)	3.08 (0.07‐21.49)	0.26
Unknown	6 (0.5)	0 (0.0)			1 (3.0)			0 (0.0)		
Lymphovascular space involvement										
No/unknown	964 (79.3)	49 (72.1)	1.00		24 (72.7)	1.00		20 (74.1)	1.00	
Yes	251 (20.7)	19 (27.9)	1.49 (0.81‐2.63)	0.15	9 (27.3)	1.44 (0.58‐3.26)	0.36	7 (25.9)	1.34 (0.47‐3.35)	0.40

a Case‐case comparisons using 2x2 tables as described in the Methods using cases with no prior cancer are the reference group.

b Other Epithelial includes mixed subtypes where serous or clear cell component does not reach % noted, or where histology was unknown (2 individuals diagnosed by curette).

**Table 4:** Overall and EC‐specific survival in patients reporting a prior cancer

Patient subgroup	Overall survival^a^			EC‐specific survival^b^		
	N Died (%)	HR^c^ (95% CI)	*P*	N Died (%)	HR^c^ (95% CI)	*P*
Patients with no prior cancer (n = 1215)	153 (12.6)	1.00		104 (8.6)	1.00	
Patients with prior breast cancer and tamoxifen use (n = 68)	23 (33.8)	1.96 (1.24‐3.11)	0.004	17 (25.0)	1.91 (1.11‐3.29)	0.02
Patients with prior breast cancer and no tamoxifen use (n = 33)	7 (21.2)	1.14 (0.46‐2.78)	0.8	4 (5.9)	1.33 (0.49‐3.64)	0.6
Patients with prior LS‐associated cancers (n = 27)	3 (11.1)	0.72 (0.23‐2.28)	0.6	1 (1.5)	0.33 (0.05‐2.39)	0.3

a Survival time estimated from date of primary treatment for EC to date of death from any cause censored.

b Survival time estimated from date of primary treatment for EC to date of death due to EC.

c Effect estimates are based on Cox models adjusted for age at EC diagnosis, FIGO stage, tumor subtype, and LVSI.

### 3.4 Differences in EC tumor and germline MMR status

The relationship between MMR status and prior cancer is summarized in Table 5. EC patients with a proven germline pathogenic MMR gene variant were significantly more likely to report a prior cancer than cases whose cancers were MMR proficient (45.5% vs 14.3%, *P* = 0.0001). Prior cancers reported by pathogenic MMR gene variant carriers included breast cancer with tamoxifen use (9.1% vs 6.1% in the reference group, *P* = 0.6), prior LS‐associated cancer (18% vs 2%, *P* < 0.0001), and other prior cancer (18.2% vs 4.7%, *P* = 0.005). Patients with MMR tumor deficiency but no MMR germline pathogenic variant identified were slightly more likely to report no prior cancer compared to the reference group of MMR proficient cases, but this difference was not statistically significant (91.5% vs 85.7%, *P* = 0.07).

**Table 5:** MMR status according to patient prior cancers

Patient subset with IHC data	MMR proficient^a^ n = 558	MMR deficient (no pathogenic variant)^b^ n = 142	*P* ^†^	MMR deficient (pathogenic variant carrier)^c^ n = 22	*P* ^†^
	N (%)	N (%)		N (%)	
No prior cancer	478 (85.7)	130 (91.5)	0.07	12 (54.5)	0.0001
Prior breast cancer with tamoxifen use	34 (6.1)	5 (3.5)	0.20	2 (9.1)	0.60
Prior breast cancer—no tamoxifen use	11 (2.0)	2 (1.4)	0.60	0 (0.0)	na
Prior Lynch cancer	11 (2.0)	2 (1.4)	0.60	4 (18.2)	<0.0001
Patients with dother prior cancer	26 (4.7)	4 (2.8)	0.30	4 (18.2)	0.005

a Mismatch repair (MMR) proficient patients (n = 558) were proficient in immunohistochemistry testing for EC tumor expression of MLH1, MSH6 and PMS2, and were not tested for gene sequence changes ‐ assumed to have no pathogenic variant.

b MMR deficient patients (n = 142) were deficient in immunohistochemistry for at least one of MLH1, MSH2, MSH6, or PMS2, and genetic testing identified no pathogenic or likely pathogenic variant. MMR deficiency was confirmed (somatic MLH1 methylation) or assumed to be due to somatic causes.

c MMR deficient pathogenic variant carriers were deficient in immunohistochemistry for at least one of MLH1, MSH2, MSH6, or PMS2, and genetic testing identified of a pathogenic MMR variant consistent with pattern of IHC loss.

d Other Prior Cancers among MMR proficient were melanoma (n = 18), thyroid (n = 2), cervix (n = 1), larynx (n = 1), other unspecified (n = 4); among MMR deficient no pathogenic variant were melanoma (n = 3) and thyroid (n = 1); among MMR‐deficient pathogenic mutation carriers were melanoma (n = 2), cervix (n = 1), and thyroid (n = 1).

^†^ *P*‐values are derived from chi‐squared comparison of proportions between MMR deficient and proficient cases.

## 4 DISCUSSION

We hypothesized that there may be a genetic contribution to EC risk in patients reporting prior cancer, after accounting for tamoxifen treatment for prior breast cancer. Our baseline comparison of reported prior cancer in controls compared to EC cases supported the known association between tamoxifen treatment for breast cancer and subsequent risk of EC. There was no evidence to suggest that any reported prior cancer, reported breast cancer without tamoxifen treatment, or LS‐associated cancers combined, was more common for cases compared to controls, confirming that reported prior cancer alone is not useful as a generic feature to identify women at increased risk of suspected familial EC.

Recognizing that sample sizes for the subgroups of women with prior cancer were small, our results nonetheless suggest that epidemiological factors conferring increased risk of EC have similar directions of effect for EC cases with no prior cancer compared to those with prior breast cancer or LS‐associated cancer. We considered explanations for the possible exceptions to anticipated associations with epidemiological risk factors for women with prior cancer, noting that in all instances the relevant risk estimates included unity. Smoking is thought to reduce circulating endogenous estrogen and so result in a reduced risk of EC, as observed for EC after prior LS‐associated cancer or prior breast cancer in the absence of tamoxifen use. The observation that smoking was not associated with EC among women with prior breast cancer and tamoxifen use might be explained by the fact that exposure to tamoxifen and not endogenous estrogen is the major mechanism for risk in this subset of women. There is no obvious explanation for the observation that women with prior breast cancer and no tamoxifen use did not show an inverse association with increasing age at menarche, especially since this subgroup of women did show a statistically significant inverse EC risk associated with increasing parity. It thus seems reasonable to discount the observation as spurious, reflecting the small sample size for this group.

All subgroups of individuals with prior cancer showed an increased proportion of reported family history of cancer (FDR and/or SDR) compared to EC cases with no prior cancer or controls with no prior cancer, even those with tamoxifen‐treated breast cancer (Table 2). EC risk associated with reported family history of breast cancer did not differ according to tamoxifen usage for prior breast cancer (OR 1.98 for prior breast cancer with tamoxifen use vs OR 1.95 for prior breast cancer no tamoxifen use). Interestingly, for patients with prior LS‐associated cancer, the elevation in EC risk for reported family history of LS‐associated cancer (OR 2.34; 95% CI 1.05‐5.09) was similar to that for reported family history of breast cancer (OR 2.15; 95% CI 0.99‐4.69). These observations are consistent with the expectation that individuals with genetic factors underlying their disease might present with prior cancer and/or family history of cancer. Indeed, all patients with a prior LS‐associated cancer reported a family history of cancer, and the cancer type in relative/s was designated as LS‐associated for 17/27 (63%) of these patients (Table 2).

Report of prior LS‐associated cancer is considered an indicator of MMR pathogenic variant carrier status.^29^ Patients identified as carriers of a pathogenic MMR gene variant were overall more likely to report a prior cancer. Recognizing that these observations were based on a small sample set of 22 carriers, it was surprising to note that less than half of the prior cancers reported (4/10 total) were LS‐associated cancers, with the remainder comprising tamoxifen‐treated breast carcinoma (n = 2), melanoma (n = 2), and cervical and thyroid carcinomas. Nonetheless, when considering MMR status within groups defined by prior cancer, 4/17 (23.5%) individuals with prior LS‐associated cancer were identified to be a pathogenic variant carrier, compared to 12/620 (1.9%) of individuals with no prior cancer. This observation emphasizes that diagnosis of a first primary LS‐associated cancer is an important clinical indicator to prioritize MMR gene testing and could enable timely identification of MMR gene pathogenic variant carriers for implementation of appropriate cancer risk reduction strategies to prevent second primary cancers. However, it is also important to note that more than half (54.5%) of MMR pathogenic variant carriers did not report any prior cancer, indicating that history of prior LS‐associated cancer alone has poor sensitivity to delineate patients with EC due to a germline MMR gene defect.

Although tamoxifen has clearly been shown to be beneficial for treatment of breast cancer,^30^, ^31^ it is associated with a two‐ to threefold increased risk of EC compared to age‐matched women in the general population.^5^, ^8^, ^32^ There is evidence that this subgroup of EC patients are enriched for poor‐prognosis pathological features and/or have poor survival,^6^ of interest since tamoxifen users might be expected to be under greater scrutiny for symptoms of EC. A brief review summarizing the main findings of selected studies is presented in Table S2. As detailed in Table 3, our findings support previous reports that EC after tamoxifen use for breast cancer has adverse prognostic features. In our study, the proportion of MMMTs after tamoxifen treatment for breast cancer was increased (13% vs <3% in cases with no prior cancer), equating to a 5.6‐fold increase. Patients were more likely to present with stage IV EC after tamoxifen treatment for breast cancer (9% vs 3% for tamoxifen nonusers). Interestingly, these correlations appear to be independent; only two of the nine individuals with MMMT (22.2%) were Stage IV and another 3 (33.3%) were stage III. Our study did not collect information on duration of tamoxifen use, so we were not able to assess if MMMT are more likely to arise after long‐term tamoxifen use (as previously reported). Compared to EC patients with no prior cancer, women diagnosed with EC after tamoxifen‐treated breast cancer had significantly worse survival outcomes (HR 1.96; *P* = 0.004 any cause, HR 1.91; *P* = 0.02 EC‐specific). In stark contrast, there was no evidence for a survival difference for women reporting prior breast cancer without tamoxifen treatment or prior LS‐associated cancers (*P* ≥ 0.3). Notably, although women with prior tamoxifen‐treated breast cancer had a higher prevalence of MMMT tumors compared to those with prior breast cancer and no tamoxifen use, this survival difference was not totally accounted for by poorer prognostic features of EC subtypes (Table 4).

A limitation of our study was that information on prior cancers was based on patient self‐report, although where possible these were confirmed using data from clinical follow‐up, and pathology reports. Information appeared to be reasonably accurate with the exception of self‐reported prior ovarian cancer, most of which were determined to be concurrent with EC. ANECS participants had to have an intact uterus at the time of EC diagnosis to participate, so it is not surprising that there were so few prior ovarian cancers among EC cases since standard surgical management for ovarian carcinoma, which accounts for the great majority of ovarian cancers, includes hysterectomy. Clinical follow‐up was undertaken for cases but not controls, allowing us to verify self‐reported data for EC cases only. Since we restricted the reference group to controls with no prior cancer for case‐control analysis, failure to identify over‐report of prior cancers in this group (estimated to be only 2/740 controls based on observations for cases) would have minimally biased our overall results toward the null. Further, any bias in data quality should not have impacted case‐case analysis.

This study has confirmed the relationship between EC risk and prior tamoxifen‐treated breast cancer, and also previous reports that tamoxifen‐treated breast cancers are more likely to lead to poorer prognosis EC. However, considering all possible sources of information regarding tamoxifen treatment, we also estimate that one‐third of all EC after breast cancer did not appear to be tamoxifen related. Epidemiological risk factors were largely similar across subgroups according to prior cancer report. Supporting our hypothesis that cancer‐causing genetic factors will be enriched in individuals with prior cancer, a family history of cancer was increased for all patient subgroups reporting prior cancer. Surprisingly, this included family history of LS‐associated cancers for EC cases with tamoxifen‐treated prior breast cancer, and family history of breast cancer for individuals with prior LS‐associated cancer. Further, while prior LS‐associated cancer is clearly an indicator of MMR pathogenic variant status, so is report of other prior cancer types. However, 55% of established carriers did not report prior cancer, supporting the increasingly accepted role of universal immunohistochemical or molecular analysis of all ECs at diagnosis to detect patients with LS.

An important finding arising from this study is that women developing EC after tamoxifen treatment for breast cancer exhibited poorer survival—even after adjustment for known EC‐related prognostic features. We suggest that it is important to ensure longer‐term monitoring of women treated with tamoxifen for symptoms of EC. We also believe there is scope for studies assessing the biological mechanism underlying the poorer prognosis of this subset of EC patients.

## CONFLICT OF INTEREST

None declared.
