# Supplementary figures and images for: Risk and prognostic factors for endometrial carcinoma after diagnosis of breast or Lynch‐associated cancers—A population‐based analysis
Source: Cancer Med. 2018 Nov 28;7(12):6411–22. doi: 10.1002/cam4.1890 (PMC6308118; doi:10.1002/cam4.1890)

**A**

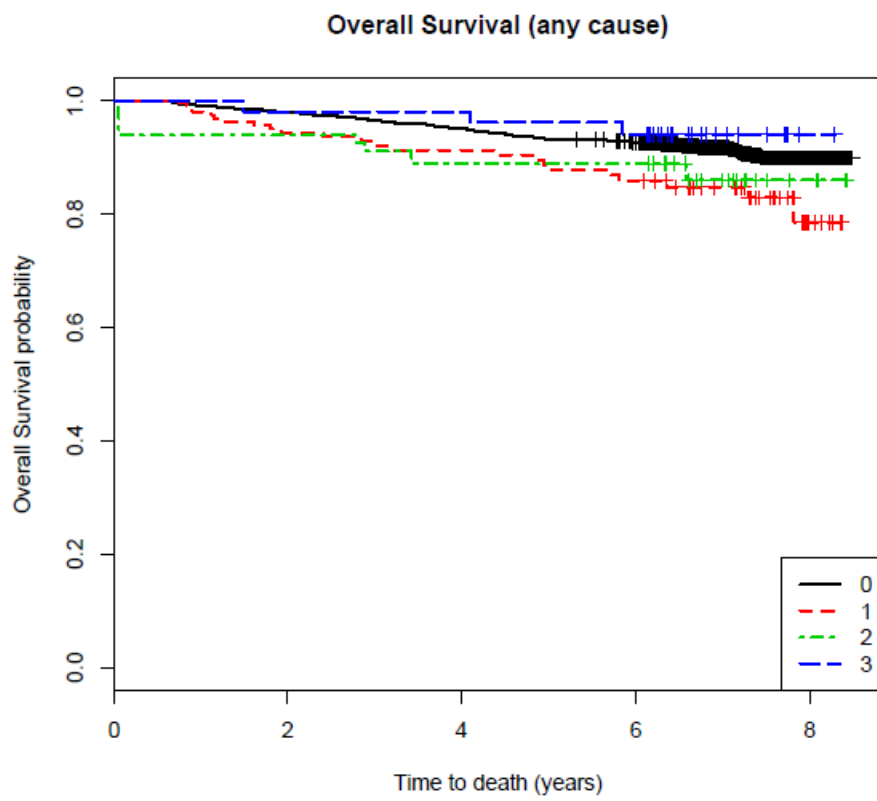

**B**

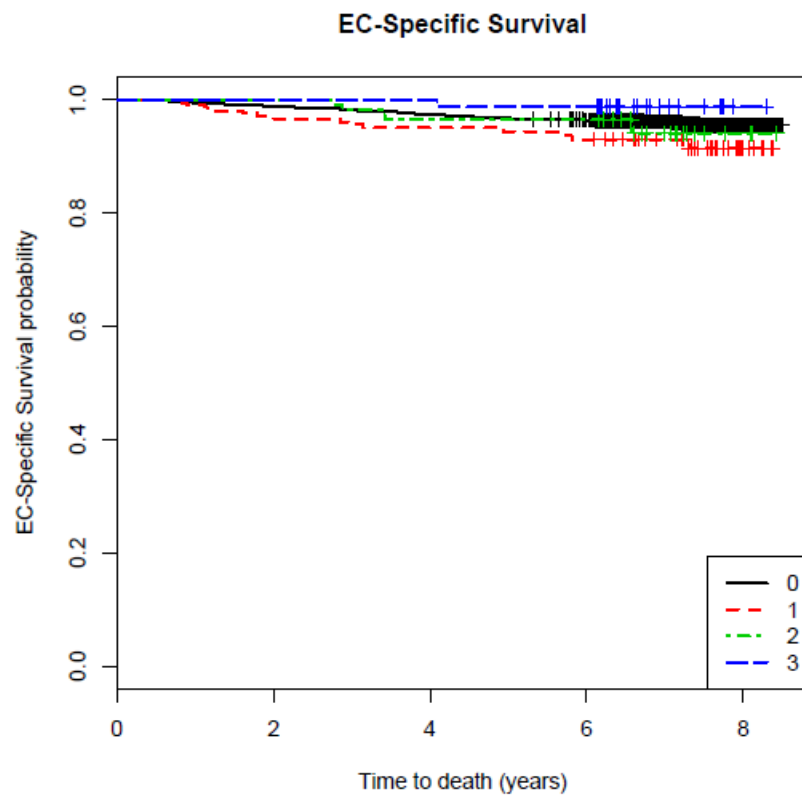

Supplement: Supplementary file 1 [file CAM4-7-6411-s001.pdf]
