# Supplementary material for: Risk and prognostic factors for endometrial carcinoma after diagnosis of breast or Lynch‐associated cancers—A population‐based analysis
Source: Cancer Med. 2018 Nov 28;7(12):6411–22. doi: 10.1002/cam4.1890 (PMC6308118; doi:10.1002/cam4.1890)
Supplement: Supplementary file 2 [file CAM4-7-6411-s002.docx]

Table S1: Prior cancer reported in patients and controls —overall proportion, and separated by cancer type (breast, LS-associated, other)

|  | **Cases (n=184 of 1399)** | |  | **Controls (n=86 of 740)** | |  | **^b^ P** | |
| --- | --- | --- | --- | --- | --- | --- | --- | --- |
| **Prior Cancers** | **^a^N Prior cancers reported** | **Proportion of all cases (N=1399)** | **Report of Additional Prior Cancers** | **^a^N Prior cancers reported** | **Proportion of all Controls (N=740)** | **Report of Additional Prior Cancers** |  | |
| **Overall - Women with any Prior Cancer** | **184** | **0.132** |  | **86** | **0.116** |  | 0.3 | |
| **Breast Cancer** | **101** | **0.072** | 8 individuals in total reported other prior cancers (see details below) | **41** | **0.055** | 2 individuals reported other prior cancers, one colon and one ovarian cancer | 0.1 | |
| Tamoxifen-treated | 68 | 0.049 | 4 individuals reported other cancers; 1 reported colon and unspecified leukemia; 1 reported malignant melanoma; 1 reported lymphoid leukemia; 1 reported melanoma in situ | 21 | 0.028 |  | 0.02 | |
| No/unknown tamoxifen treatment | 33 | 0.024 | 4 individuals reported other cancers; 1 reported stomach cancer and melanoma; 2 reported colon cancer; 1 reported malignant melanoma; 1 reported 'other immunoproliferative disease' | 20 | 0.027 |  | 0.7 | |
| **Lynch Syndrome Associated Cancer** | **27** | **0.019** |  | **15** | **0.020** |  | 0.9 | |
| Ovarian | 2 | 0.001 | 1 also reported melanoma in situ and unspecified lymphoma | 1 | 0.001 | also reported breast cancer |  | |
| Colon | 19 | 0.014 | 2 also reported breast cancer no tamoxifen; 1 also reported breast cancer with tamoxifen & unspecified leukemia; 2 also reported malignant melanoma | 9 | 0.012 | 1 also reported breast cancer, & 2 reported melanoma |  | |
| Stomach | 2 | 0.001 | 1 also had breast cancer no tamoxifen & malignant melanoma | 0 | 0.000 |  |  | |
| Brain/Meninges | 2 | 0.001 |  | 0 | 0.000 |  |  | |
| Bladder | 0 | 0.000 |  | 2 | 0.003 | 1 reported thyroid cancer |  | |
| Renal Pelvis | 2 | 0.001 |  | 1 | 0.001 |  |  | |
| Ill-defined digestive organ | 0 | 0.000 |  | 1 | 0.001 |  |  | |
| Small intestine | 0 | 0.000 |  | 1 | 0.001 |  |  | |
| **Other Cancer Type** | **68** | **0.049** |  | **38** | **0.051** |  | **0.8** | |
| Thyroid | 5 | 0.004 |  | 2 | 0.003 | 1 reported bladder cancer |  | |
| Cervix - uteri | 7 |  | 1 Person >50 also had Malignant neoplasm of kidney, except renal pelvis | 5 | 0.007 | 1 also had neoplasm of vagina <50 |  | |
| Melanoma | 41 | 0.029 | 2 also reported colon cancer, 1 reported stomach & breast cancer no tamoxifen, 2 reported had breast cancer with tamoxifen, 1 reported breast cancer no tamoxifen | 19 | 0.026 | 2 reported colon , and 1 had neoplasm of the vagina | |  |
| Lip, oral cavity, tongue, pharynx, larynx | 2 | 0.001 |  | 1 | 0.001 |  |  | |
| NHL/unspecified lymphoma/other immunoproliferative disease | 6 | 0.004 | 1 also reported a ovarian cancer (true prior) & 1 also reported thyroid cancer | 3 | 0.004 | 1 also had lymphoid leukemia |  | |
| Vagina neoplasm | 0 | 0.000 |  | 1 | 0.001 | also had melanoma <50 |  | |
| Malignancy ill-defined | 1 | 0.001 |  | 1 | 0.001 |  |  | |
| Leukemia/lymphoid or unspecified | 4 | 0.003 | 1 reported prior bowel & breast cancer with tamoxifen; 1 had prior breast cancer with tamoxifen | 0 | 0.000 |  |  | |
| Kidney except renal pelvis | 2 | 0.001 | 1 also reported cancer of the cervix | 0 | 0.000 |  |  | |
| Placenta | 1 | 0.001 |  | 0 | 0.000 |  |  | |
| Malignant neoplasm of eye and adnexa | 1 | 0.001 |  | 0 | 0.000 |  |  | |
| Other unspecified | 5 | 0.004 | 1 also reported breast cancer no tamoxifen, 1 reported cancer of the larynx, 1 reported leukemia | 3 | 0.004 |  |  | |

^a^Ns for reported cancers will not sum to N women with prior cancer (184 patients and 86 controls) since some women reported multiple prior cancers. 3 Melanoma in-situ excluded from counts of 'Other cancers'

^b^P-value derived from chi-squared comparison of proportions between patients and controls

Table S2: Summary of selected studies reporting increased risk of rare but aggressive carcinosarcoma/MMMT among women treated with tamoxifen

| **Reference** | **No. of Patients/controls** | **Study details** | **Age (range in years)/menopausal status** | **Tamoxifen dose and/or duration (range)** | **Main finding** |
| --- | --- | --- | --- | --- | --- |
| Curtis et al^1^ | 39,451 breast cancer patients designated as tamoxifen users/ 67,190 breast cancer survivors considered non-tamoxifen users | SEER study (1980 - 2000); patients received hormones as their first course of treatment; previous experience was that >90% were treated with tamoxifen, so all patients designated as tamoxifen users | Breakdown for age at diagnosis of breast cancer; the highest MMMT O/E ratio was for the 60-69 age group | NA | Risk of EC was >2-fold for women treated with tamoxifen. Increased risk of MMMT uterine tumors was >4-fold for prior breast cancer on tamoxifen, but 38% for non-tamoxifen. Tamoxifen users who survived ≥5 years had an 8-fold increased risk of carcinosarcoma/MMMT. Cumulative mortality from EC among tamoxifen users was very low (0.37%) 15 years post treatment. After diagnosis of breast cancer carcinosarcoma/MMMT tended to be detected later (median time to diagnosis = 7.5 years). There was no difference in risk of clear cell adenocarcinoma between tamoxifen users vs non-users. Considering cancers of the endometrium alone, the proportion of MMMT was 9.7% in this study. |
| Swerdlow et al^2^ | 813 women with breast cancer prior to EC diagnosis/1067 controls who had breast cancer but no subsequent EC | A population-based British cancer registry study; aim was to assess relationship between tamoxifen and other risk factors for EC. | ≥55 age at diagnosis for breast cancer for both cases and controls | Median duration was 11 years. Results stratified by average daily dose of tamoxifen, from 10 mg to 40 mg | Patients ever treated with tamoxifen had a 13-fold increased risk of carcinosarcoma/MMMT tumors and sarcomas [OR=13.5 (4.1–44.5)], compared to a 2-fold increased risk of adenocarcinoma [OR=2.1 (1.6–2.7)] and a 3-fold increased risk of clear cell and serous tumors [OR=3.1 (0.8–17.9)] They report a significant trend in increased risk for longer duration of tamoxifen. |
| Bergman et al^3^ | 309 women with breast cancer prior to EC/860 matched controls with breast cancer and no EC | Dutch case control study of tamoxifen use in 299/309 breast cancer cases and 245/860 controls. |  | long-term use considered ≥2 years use | Long-term tamoxifen use was associated with higher likelihood of carcinosarcoma/MMMT (15.4% among users vs 2.9% of non-users). EC stages III & IV was more frequent among tamoxifen users than non-users (17.4% vs 5.4%). EC-specific survival was significantly worse in women who used tamoxifen for at least five years. |
| Ngo et al^4^ | 363 EC patients | Patients grouped according to 1) no prior breast cancer (n=283); 2) prior breast cancer without tamoxifen (n=37) 3) prior breast cancer with tamoxifen (n=43) | range 24–93; median was 65; 91% were menopausal | NA | There was a non-significant greater proportion of carcinosarcomas in group with prior breast cancer with tamoxifen (11.7% vs 5.4% in group 2 & 4.2% in group 1). Group 3 had shorter median overall survival (94 months) vs group 2 (120 months) vs group 1 (180 months) (p=0.0006). |
| Tergas et al^5^ | 115 patients with high grade EC; 15 had tamoxifen | Comparison of clinico-pathologic variables for surgically staged patients with grade 3 EC tumors based on tamoxifen exposure | Mean age at EC diagnosis was 73 (tamoxifen users) and 67 (non-tamoxifen users) | NA | 60% of tamoxifen patients had carcinosarcoma/MMMT compared to 30% among those who did not have tamoxifen. Overall survival was shorter form those who had tamoxifen vs those who did not (16.6 vs 32.2 months). |

**References:**

1. Curtis RE, Freedman DM, Sherman ME, Fraumeni JF, Jr. Risk of malignant mixed mullerian tumors after tamoxifen therapy for breast cancer. *Journal of the National Cancer Institute* 2004;**96**: 70-4.

2. Swerdlow AJ, Jones ME, British Tamoxifen Second Cancer Study G. Tamoxifen treatment for breast cancer and risk of endometrial cancer: a case-control study. *Journal of the National Cancer Institute* 2005;**97**: 375-84.

3. Bergman L, Beelen ML, Gallee MP, Hollema H, Benraadt J, van Leeuwen FE. Risk and prognosis of endometrial cancer after tamoxifen for breast cancer. Comprehensive Cancer Centres' ALERT Group. Assessment of Liver and Endometrial cancer Risk following Tamoxifen. *Lancet* 2000;**356**: 881-7.

4. Ngo C, Brugier C, Plancher C, de la Rochefordiere A, Alran S, Feron JG, Malhaire C, Scholl S, Sastre X, Rouzier R, Fourchotte V, Gynecological Cancer Study Group of Institut C. Clinico-pathology and prognosis of endometrial cancer in patients previously treated for breast cancer, with or without tamoxifen: a comparative study in 363 patients. *Eur J Surg Oncol* 2014;**40**: 1237-44.

5. Tergas AI, Buell-Gutbrod R, Gwin K, Kocherginsky M, Temkin SM, Fefferman A, Lengyel E, Yamada SD. Clinico-pathologic comparison of type II endometrial cancers based on tamoxifen exposure. *Gynecologic oncology* 2012;**127**: 316-20.

Figure S1: Legend

**Figure S1: Overall Survival of EC according to prior cancer status**

Kaplan Meier curves for death due to EC (A) and death from any cause (B) according to prior cancer status; 0 (solid black line) = EC patients reporting no prior cancer (n=1215); 1 (red dashed line) = EC patients reporting prior breast cancer with tamoxifen treatment (n=68); 2 (green dash-dot-dash line) = EC patients reporting prior breast cancer without tamoxifen treatment (n=33); 3 (blue long dash line) = EC patients reporting prior Lynch-associated cancers (n=27). KMs are based from Cox models adjusted for age at EC diagnosis (continuous), tumor stage (entered as factor variables for FIGO I, II, and III + IV), tumor subtype (entered as factor variables for endometrioid Grade 1, endometrioid Grade 2, endometrioid Grade 3, serous, clear cell carcinoma, carcinosarcoma/MMMT, other epithelial), and LVSI (no/unknown vs yes).
